# Radicicol-Mediated Inhibition of Topoisomerase VIB-VIA Activity of the Human Malaria Parasite *Plasmodium falciparum*

**DOI:** 10.1128/mSphere.00025-15

**Published:** 2016-01-06

**Authors:** Sureshkumar Chalapareddy, Swati Chakrabarty, Mrinal Kanti Bhattacharyya, Sunanda Bhattacharyya

**Affiliations:** a Department of Biotechnology and Bioinformatics, School of Life Sciences, University of Hyderabad, Hyderabad, India; b Department of Biochemistry, School of Life Sciences, University of Hyderabad, Hyderabad, India; The Hebrew University

**Keywords:** *Plasmodium* topoisomerase VI, *Plasmodium* type IIB topoisomerase, radicicol, PfTopoVIB

## Abstract

*Plasmodium falciparum* topoisomerase VIB (TopoVIB)-TopoVIA (TopoVIB-VIA) complex can be potentially exploited as a drug target against malaria due to its absence from the human genome. Previous work in our laboratory has suggested that *P. falciparum* TopoVIB (PfTopoVIB) might be a target of radicicol since treatment of parasite cultures with this antibiotic is associated with upregulation of *Plasmodium* TopoVIB at the transcript level as well as at the protein level. Further studies demonstrated that radicicol treatment impaired mitochondrial replication of human malaria parasite *P. falciparum*. However, the technical challenge associated with the expression of the above protein complex hampered its functional characterization. Using *Saccharomyces cerevisiae* as a heterologous system, we expressed PfTopoVIB (Myc-tagged) and PfTopoVIA (Flag-tagged) (PfTopoVIB-VIA) proteins. Yeast two-hybrid analysis showed the formation of PfTopoVIB homodimers and PfTopoVIB/PfTopoVIA heteromers. Our study demonstrated that PfTopoVIB and PfTopoVIA together can rescue the lethal phenotype of yeast *ΔtopoII* mutants, whereas *Plasmodium* topoisomerase VIB alone cannot. Using yeast cell-free extracts harboring the PfTopoVIB-VIA protein complex, we have performed a decatenation assay and observed that PfTopoVIB-VIA can decatenate DNA in an ATP- and Mg^2+^-dependent manner. The specificity of this enzyme is established by abrogation of its activity in the presence of PfTopoVIB-specific antibody. Our study results show that radicicol and etoposide can specifically inhibit PfTopoVIB-VIA decatenation activity whereas the gyrase inhibitor novobiocin cannot. Such a yeast-based assay system can be employed in screening specific inhibitors against *Plasmodium* VIB-VIA.

**IMPORTANCE** In this study we characterize topoisomerase VI from *Plasmodium falciparum* using genetic and biochemical approaches. We use various inhibitors and identify radicicol as a specific inhibitor of its decatenation activity. We establish a very simple and economical biochemical assay system that can be exploited to screen inhibitors of PfTopoVI.

## Abstract (precis)

In this study we characterize topoisomerase VI from *Plasmodium falciparum* using genetic and biochemical approaches. We use various inhibitors and identify radicicol as a specific inhibitor of its decatenation activity. We establish a very simple and economical biochemical assay system that can be exploited to screen inhibitors of PfTopoVI.

## INTRODUCTION

According to the World Malaria Report 2014 (21), about 3.3 billion people, representing almost half of the total world population, are presently at risk of malaria. The main victims of this disease are children under the age of 5. Over the past years, *Plasmodium falciparum* developed multiple drug resistance and hence there is an urgent need to discover the new target molecules which are crucial for parasite survivability.

Malaria parasite experiences three developmental stages, namely, the ring, trophozoite, and schizont stages, during its asexual replication within human red blood cells (RBC). In the schizont stage, the parasites undergo multiple nuclear replications without cytoplasm division. This kind of cell division, namely, endoreduplication, leads to a rapid increase in pathogen biomass which directly correlates with disease severity. Endoreduplication commonly occurs in plants. It has been established in *Arabidopsis thaliana* that *HYP6*, which encodes *A. thaliana* TopoVIB (AtTopoVIB), and *RHL2*, which encodes AtTopoVIA, are the essential components required for decatenation of the replicated chromosome during endoreduplication (1). The *Plasmodium* genome sequence shows the presence of putative PfTopoVIB and PfTopoVIA (2). However, until now, there has been no report illustrating the biochemical properties of these enzymes.

Topoisomerases are broadly classified into two types (type I and type II) on the basis of their differences in structure and function (3). Type I topoisomerase cleaves one strand of duplex DNA and then reseals it in an ATP-independent manner. It plays a critical role in DNA replication and transcription by acting as a swivel and thereby smoothing the passage of DNA polymerase and RNA polymerase along the DNA. Type II topoisomerase is primarily involved after DNA replication during separation of daughter strands. It cleaves both strands of DNA and joins them with the help of ATP hydrolysis and thereby allows decatenation of DNA. They bind at the 5′ end of the broken DNA, generating a 5′ phosphotyrosyl linkage and a free 3′ hydroxyl group at the broken junction.

The malaria parasite *P. falciparum* encodes topoisomerase I, II, III, and VI and gyrase. *Plasmodium* gyrase has been extensively characterized (4) and is observed to play an important role in apicoplast replication (5). PfTopoI (6) and PfTopoII (7) have also been characterized biochemically, and several specific inhibitors of their activity have been reported.

Topoisomerase VI is a type IIB topoisomerase which was first identified in *Sulpholobus shibatae*, an archaeal species (8). It has two subunits, topoisomerase VIA and VIB. The active form of the enzyme consists of the heterotetramer (A_2_B_2_). The crystal structure of *S. shibatae* topoisomerase VIB reveals the presence of ATP binding domain, H2TH (helix 2 turn helix) domain, and transducer domain (9). H2TH is not observed in other topoisomerases, and its function is not clearly understood. The transducer domain mediates communication between the N-terminal clamp and the C-terminal domain (10), and it also interacts with the N terminus of TopoVIA (9). Structural studies revealed that there are striking similarities between the ATP binding domains of TopoVIB and that present in the N-terminal domain of GHKL (gyrase-Hsp90-CheA histidine kinase-MutL) ATPases and topoisomerase II. They all share a small three-dimensional fold within the ATPase domain known as the Bergerat fold. X-ray crystallographic data show that radicicol, an antifungal antibiotic which was originally isolated from the fungus *Monosporium bonorden*, binds to the ATP binding pocket of topoisomerase VIB (11) and inhibits the dimerization of TopoVIB ATPase domain and competitively inhibits ATP hydrolysis. However, members of the coumarin group of drugs such as novobiocin which can inhibit gyrase fail to interact with TopoVIB (8).

TopoVIA is structurally similar to the TOPRIM (topoisomerase-primase) domain present in type IIA topoisomerases which is responsible for DNA cleavage. The TOPRIM fold contains two small conserved motifs that have three invariant acidic residues (one glutamate and two aspartates). Glutamate plays a role in the strand-rejoining activity of TopoVIA, whereas the conserved aspartate coordinates with the magnesium ion that is essential for the activity of TOPRIM domain-containing proteins (12). TopoVIA also shares significant similarity with Spo11, a protein which plays an important role in creating DNA double-strand breaks during meiotic recombination. TopoVIA possesses another domain, namely, the CAP domain, which has a characteristic helix-turn-helix fold which is present in bacterial DNA binding catabolite activator proteins. It contains the conserved tyrosine residue which is important for the DNA cleavage.

Though the *Plasmodium* genome sequence was available more than a decade ago, the challenge in expressing PfTopoVI in a heterologous expression system impeded its functional characterization. We have used *Saccharomyces cerevisiae* as a surrogate system to characterize the biochemical properties of PfTopoVI. As overexpression of topoisomerase II is toxic to the yeast cell survival, we have used a conditional expression system to induce PfTopoVI for a short period of time and performed its biochemical characterization using the cell extract. Our study results demonstrate that *P. falciparum* topoisomerase VIB and VIA (PfTopoVIB-VIA) can together support the growth of Δ*topoII*-null yeast cells, which is otherwise lethal. The results of our earlier study showed that radicicol docks into the Bergerat fold present in the ATPase domain of PfTopoVIB (13). We observed that radicicol can arrest schizont development of parasite in a reversible manner and that it inhibits the mitochondrial replication of the parasite (13). We speculated that PfTopoVIB might be the target of radicicol. In this paper, we report that we have demonstrated that radicicol inhibits the decatenation activity of PfTopoVI, which supports our earlier observation. This yeast-based assay system is extremely economical and can be employed in future to study different chemically modified forms of radicicol. This system can also be used to screen new inhibitors of PfTopoVI.

## RESULTS

### Sequence analysis and cloning of topoisomerase VIB and VIA of *P. falciparum*.

**FIG 1:**
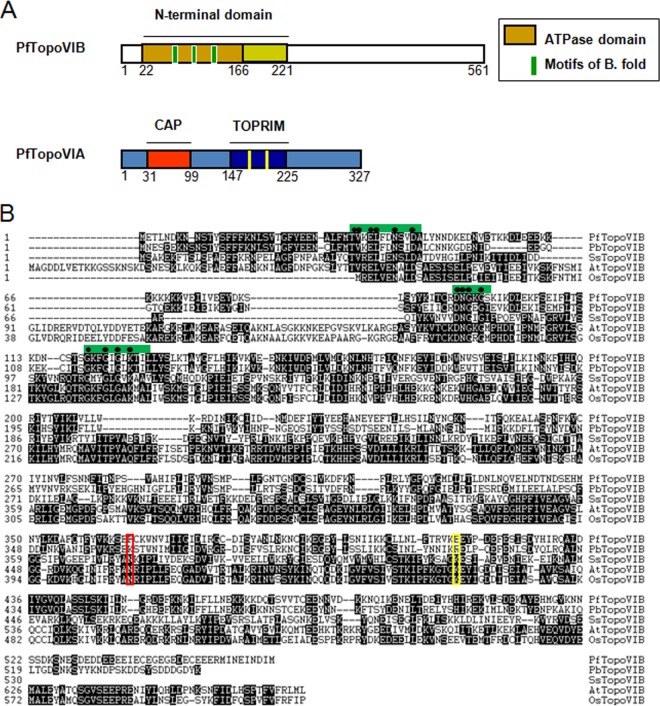

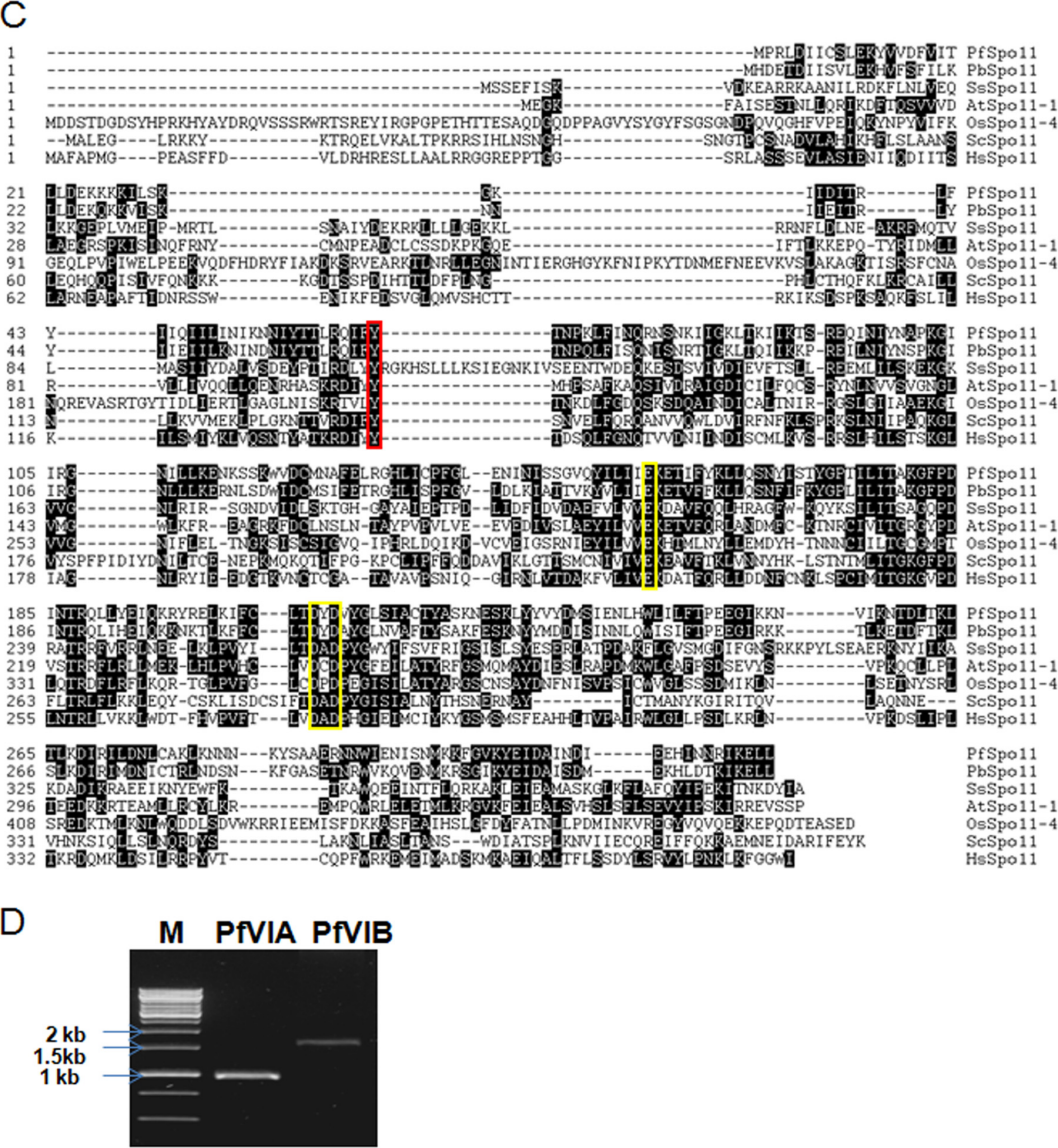
Domain organization and amplification of *Plasmodium* topoisomerase VI. (A) Schematic representation of PfTopoVIB and PfTopoVIA subunits with their functional domains. In PfTopoVIB, amino acids 22 to 161 correspond to the ATP binding region. The conserved motifs of the Bergerat fold (B. fold) are represented by green coloring. The CAP and TOPRIM domains of PfTopoVIA are represented by red and dark blue coloring, respectively. The conserved glutamate and aspartate acids in the TOPRIM domain are represented by yellow coloring. (B) Multiple-sequence alignment of PfTopoVIB (*Plasmodium falciparum*), PbTopoVIB (*Plasmodium berghei*), SsTopoVIB (*Sulfolobus shibatae*), AtTopoVIB (*Arabidopsis thaliana*), and *Oryza sativa* TopoVIB (OsTopoVIB) shows identical amino acid sequences in three motifs constituting the Bergerat fold as represented by the green box. The conserved amino acids are marked on the top as black dots. However, the parasitic TopoVIB (both PfTopoVIB and PbTopoVIB) amino acid sequences are dissimilar from those of archaeal and plant TopoVIB with respect to the H2TH and transducer domains. The conserved asparagine residue in the transducer domain is replaced by a phenylalanine residue in *P. falciparum* (represented by the red box), and the conserved lysine residue is replaced by glutamic acid in PfTopoVIB (represented by the yellow box). (C) Multiple-sequence alignment of PfTopoVIA, PbTopoVIA, SsTopoVIA, AtTopoVIA, OsTopoVIA, and ScTopoVIA (*Saccharomyces cerevisiae*) and of HsTopoVIA (*Homo sapiens*) shows a conserved tyrosine residue present in the CAP domain (represented by a red box) and conserved glutamate and aspartate residues in the TOPRIM domain (represented by a yellow box). (D) PCR amplification of *PfTOPOVIA* and *PfTOPOVIB* genes is presented.

The *P. falciparum* genome database (http://www.plasmoDB.org) shows the presence of putative topoisomerase VIB (Gene ID PF3D7_1365600) and topoisomerase VIA (Gene ID PF3D7_1217100.1). PfTopoVIB gene has seven introns, and its coding sequence is 1,686 bp long. It codes for a 561-amino-acid-containing protein. The PfTopoVIA gene has two splice variants, PF3D7_1217100.1 and PF3D7_1217100.2. The presence of different domains of PfTopoVIB and PfTopoVIA is schematically represented (Fig. 1A). Homology searches of the PfTopoVIB protein sequence revealed high (84.5%) similarity between *P. falciparum* and *P. berghei*, but there exists very little (28.8% to 31.5%) similarity with other TopoVIB orthologs as presented in Table 1. However, the N-terminal domain (amino acids 22 to 221) shows considerable (36.3% to 44.3%) similarity among the topoisomerase VIB orthologs. The core ATP binding domain (amino acids 22 to 166) is homologous to that present in GHKL ATPases and is composed of three small motifs (B1, B2, and B3) that constitute the Bergerat fold. There exists considerable sequence homology of PfTopoVIB within these motifs across various topoisomerase VIB sequences (marked as green boxes in Fig. 1B). However, the C-terminal transducer domains that are present in other topoisomerase VIB orthologs are apparently absent in PfTopoVIB according to Pfam analysis results. The results of a multiple-sequence-alignment study reveal only 9% to 15.4% sequence identity within the transducer domains of *P. falciparum* and other TopoVIB orthologs (data not shown). The highly conserved asparagine residue (marked in red) at position 366, which serves as an anchoring element between the GHKL and the transducer domain, is also missing in PfTopoVIB. Similarly, the highly conserved lysine residue (marked in yellow) at position 416 of the transducer domains through which it contacts the γ-phosphate of bound nucleotide is also absent in PfTopoVIB.

**TABLE 1:** Similarity of PfTopoVIB to other eukaryotic TopoVIB proteins

Species	% similarity^a^				
	*Plasmodium falciparum*	*Plasmodium berghei*	*Sulfolobus shibatae*	*Arabidopsis thaliana*	*Oryza sativa*
*Plasmodium falciparum*	100	71.2 (84.5)	31.5 (44.3)	33.5 (39.4)	28.8 (36.3)
*Plasmodium berghei*		100	34.2 (44.4)	34 (41.7)	31.4 (36.8)
*Sulfolobus shibatae*			100	47.1 (46.3)	45.1 (39.7)
*Arabidopsis thaliana*				100	81.8 (81.5)
*Oryza sativa*					100

^a^ Numbers in parentheses represent % similarity within the ATP binding domain.

PfTopoVIA shows about 82.6% sequence similarity with *P. berghei* TopoVIA (PbTopoVIA) and 29.5% to 42% sequence similarity with other topoisomerase VIA proteins and meiotic recombination protein Spo11 as presented in Table 2. The homology search reveals the presence of a DNA binding CAP domain (amino acids 31 to 99) and of a TOPRIM domain (amino acids 147 to 225) in PfTopoVIA. There exists 34.1% to 42.7% sequence similarity among the CAP domains of related topoisomerase VIA subunits. The active-site tyrosine residue (amino acid 65) which initiates DNA cleavage is conserved in the CAP domain of PfTopoVIA as denoted by the red box (Fig. 1C). There is 57% to 59.5% sequence similarity within the TOPRIM domains of PfTopoVIA (Table 1 and 2). The alignment study shows the presence of two conserved motifs in the TOPRIM domain (marked in yellow): one is centered on the conserved glutamate residue (at amino acid 154), and the other one is centered on two conserved aspartate residues (DYD at amino acids 208 to 210).

**TABLE 2:** Similarity of PfTopoVIA to PbTopoVIA, SsTopoVIA, and other eukaryotic Spo11 proteins

Species	% similarity^a^						
	*P. falciparum*	*P. berghei*	*Sulfolobus shibatae*	*Arabidopsis thaliana*	*Oryza sativa*	*Saccharomyces cerevisiae*	*Homo sapiens*
*Plasmodium falciparum*	100	82.6 (85.5, 88.6)	36.9 (34.8, 57)	42 (38.5, 58.2)	29.5 (38.9, 59.3)	36.3 (42.7, 59.5)	39.6 (34.1, 59.2)
*Plasmodium berghei*		100	39.8 (33.7, 51.2)	40.7 (40.9, 56.8)	31.9 (35.2, 52.5)	37.1 (37.2, 54.1)	35.2 (42.7, 55.6)
*Sulfolobus shibatae*			100	45.9 (31.8, 70.1)	33.2 (23.3, 49.4)	35.6 (33.3, 44.9)	41.4 (34, 62.8)
*Arabidopsis thaliana*			40.7	100	33 (37.4, 58.4)	35.4 (40.8, 51.2)	51.4 (50, 74.4)
*Oryza sativa*					100	29 (28, 48.8)	38 (45.6, 57.7)
*Saccharomyces cerevisiae*						100	37.3 (45.4, 56)
*Homo sapiens*							100

^a^ The first and the second numbers in parentheses represent % similarity within the CAP domain and TOPRIM domain, respectively.

PfTopoVIB and PfTopoVIA were individually amplified from the cDNA library of strain 3D7 of *P. falciparum* (Fig. 1D) and subsequently cloned into PCR2.1TOPO (Invitrogen) vector. Sequencing of the cDNA of PfTopoVIA reveals the presence of the PF3D7_1217100.1 splice variant (data not shown) which contains seven introns and has a coding sequence that is 984 bp long. It codes for a 327-amino-acid-containing protein.

### PfTopoVIB interacts with PfTopoVIA.

It has been shown of topoisomerases VIB and VIA in *S. shibatae* that each individually forms a dimer and that the protein functions as a heterotetramer (8, 14). We wanted to investigate whether PfTopoVI is also involved in such interactions. We have used a yeast two-hybrid assay to examine the interaction between PfTopoVIB and PfTopoVIA.

**FIG 2:**
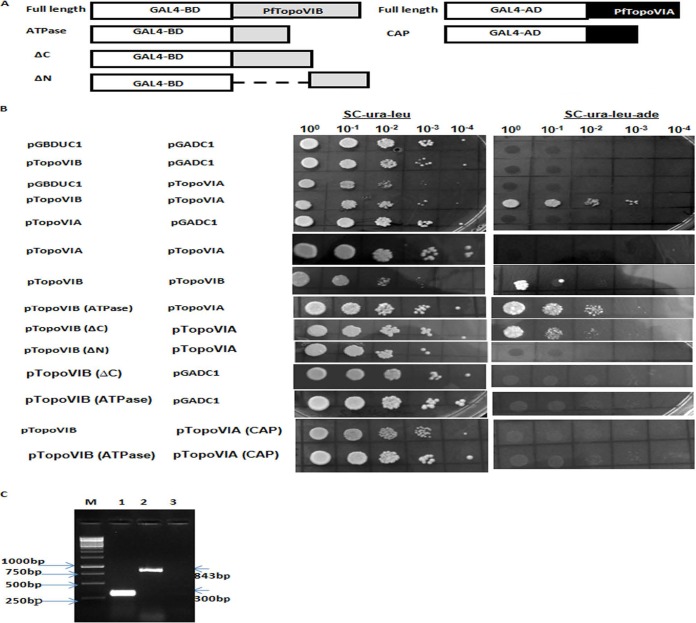
PfTopoVIB interacts with PfTopoVIA. (A) The schematic representation of full-length or truncated *PfTOPOVIB* fused to the GAL4 DNA binding domain to generate various chimeric constructs in pGBDUC1. Similarly, either full-length *PfTOPOVIA* or the CAP domain of *PfTOPOVIA* was fused to the GAL4 DNA activation domain to generate different constructs in pGADC1. (B) A yeast two-hybrid assay was performed using the PJ69-4A strain with *ADE2* as a reporter gene. Equal numbers of cells were serially diluted and spotted on the medium lacking uracil and leucine. To study the protein-protein interaction, they were spotted on the medium lacking uracil, leucine, and adenine. The left panel shows various combinations of bait and prey chimeras, and the right panel scores their interactions. (C) RT-PCR analysis shows the presence of *PfTOPOVIA* (*CAP*) (lane 1) and *PfTOPOVIB* (*ΔN*) (lane 2) transcript from respective strains harboring individual plasmids. Lane 3 serves as a negative control.

To that end, we have subcloned the full-length PfTopoVIB in bait vector as a fusion to the Gal4 DNA binding domain. Also, we have subcloned the full-length PfTopoVIA in the prey vector as a fusion to the Gal4 activation domain. The recombinant bail and prey vectors were transformed in strain PJ69-4a, and the transformed colonies were checked for interactions by measuring *ADE2* reporter gene activity, i.e., by growing the strain in the medium lacking adenine. Our results showed that PfTopoVIB or PfTopoVIA alone cannot self-activate reporter gene activity, as they fail to grow in medium lacking adenine (Fig. 2B, 2nd, 3rd, and 5th rows). However PfTopoVIB strongly interacts with PfTopoVIA as measured by their growth (Fig. 2B, 4th row) in the medium lacking adenine.

The results of our experiment also showed that PfTopoVIB can interact with itself, albeit at a lower level (Fig. 2B, 7th row). However, we found no TopoVIA self-interaction in our assay (Fig. 2B, 6th row).

We made three deletion constructs of PfTopoVIB and tested their ability to engage with PfTopoVIA. To that end, we have used the core ATPase domain present at the N-terminal domain of PfTopoVIB (amino acids 22 to 166) in our experiments. We have included in our experiments a construct containing an extended N-terminal domain (amino acids 1 to 285) of PfTopoVIB by deleting the C-terminal region (PfTopoVIBΔC). Additionally, we have made an N-terminal deletion mutant of PfTopoVIB (amino acids 280 to 561) for our analysis. The three deletion constructs of PfTopoVIB, namely, PfTopoVIB (ATPase), PfTopoVIBΔC (amino acids 1 to 285), and PfTopoVIBΔN (amino acids 280 to 561), are presented schematically in Fig. 2A. The results of our experiment showed that both PfTopoVIB (ATPase) and PfTopoVIB (ΔC) are capable of interacting with PfTopoVIA (Fig. 2B, rows 8 and 12 and rows 9 and 11, respectively). However, we did not find any growth of SKCY25 in the triple-dropout plate (Fig. 2B, row 10). In order to check whether PfTopoVIB (ΔN) is expressed in yeast, we have performed reverse transcriptase PCR (RT-PCR) analysis using cDNA isolated from strain SKCY25. Our result indicates that *PfTOPOVIBΔN* is expressed at the transcript level (Fig. 2C, lane 2). Thus, our result confirms that the C-terminal domain of PfTopoVIB (ΔN) alone fails to interact with PfTopoVIA.

We used the CAP domain of PfTopoVIA for yeast two-hybrid assays. Our experiment revealed that neither the full-length PfTopoVIB nor the ATPase domain of the same can interact with the CAP domain of PfTopoVIA (Fig. 2B, rows 13 and 14) although *PfTOPOVIACAP* is expressed at the transcript level (Fig. 2C, lane 1).

### Functional complementation of yeast topoisomerase II null mutation by *Plasmodium* PfTopoVIB-VIA.

**FIG 3:**
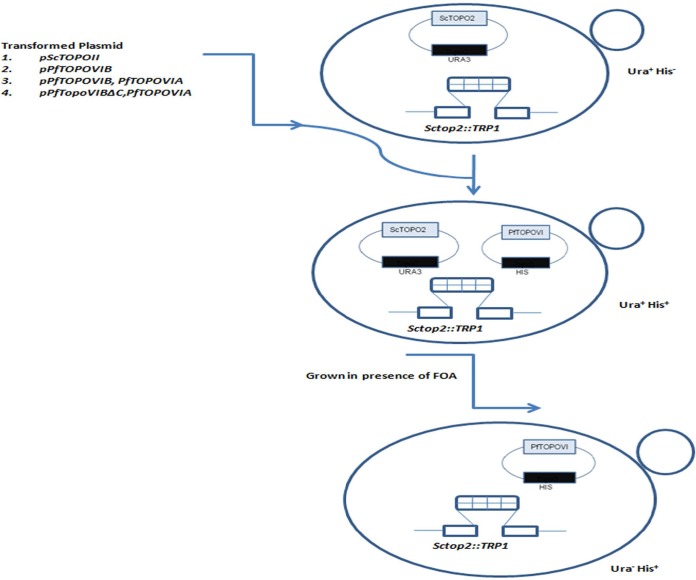
Schematic representation of genetic complementation assay in yeast. The plasmid-shuffling technique was employed to generate yeast strains harboring *PfTOPOVI*. The yeast strain was initially transformed with a *URA3*-based plasmid having *ScTOPOII*, and the chromosomal copy of yeast *TOPOII* was knocked out by *TRP1* using homologous recombination (top panel). This strain was transformed with a (*HIS3*-based) yeast expression vector carrying any of the four different genes (plasmids 1 to 4) as mentioned, resulting in Ura^+^ His^+^ cells (middle panel). Each of the transformed strains were grown on 5-fluoroorotic acid (5-FOA)-containing plates to lose the *URA3* plasmid harboring *ScTOPOII*, resulting in Ura^−^ His^+^ cells (bottom panel).

In order to decipher the *in vivo* role of PfTopoVIB-VIA, we used yeast as a surrogate system. To that end, we created a yeast *topoII* null mutant and tested whether PfTopoVI can complement yeast TopoII. Since *TOPOII* is an essential gene in yeast, we used the following strategy to create a *topoII* null mutant in yeast (Fig. 3). We transformed an expression vector (that has a *URA3* marker) harboring *S. cerevisiae TOPOII* (*ScTOPOII*) in the wild-type strain. The chromosomal *TOPOII* gene was subsequently deleted by incorporation of *TRP1* cassette using homologous recombination. The earlier expression vector harboring *ScTOPOII* was shuffled with a second vector (which is a *HIS3*-based plasmid) harboring *ScTOPOII*. The plasmid shuffling was achieved by plating the His-positive (His^+^) cells on 5-fluoroorotic acid (5-FOA). Thereby, we were able to create a cell where a chromosomal copy of *TOPOII* is deleted and *ScTOPOII* activity was maintained by an episomal *HIS3*-based plasmid. This strain has served as the positive control in our experiment.

**FIG 4:**
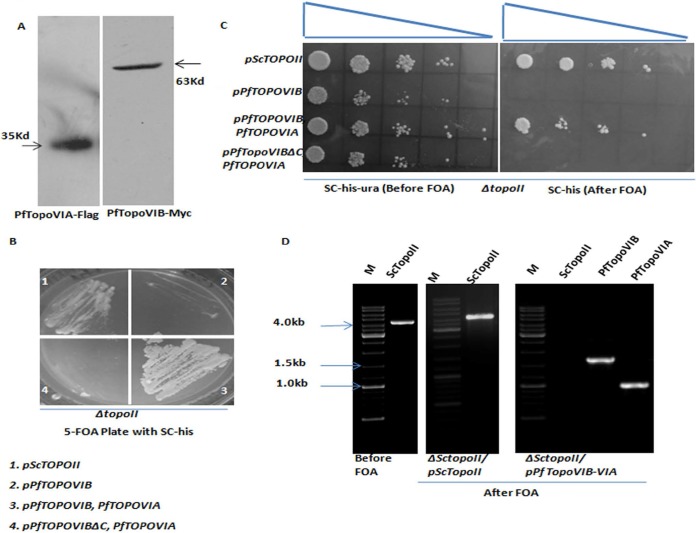
Functional complementation of yeast topoisomerase II null mutation by PfTopoVIB-VIA. (A) Western blot analysis showed the expression of Myc-tagged PfTopoVIB and Flag-tagged PfTopoVIA in *S. cerevisiae*. (B) SKCY43 (panel 1), SKCY45 (panel 2), SKCY41 (panel 3), and SKCY44 (panel 4) strains were grown in the plate containing synthetic media with 5-FOA and lacking histidine. (C) The four strains were grown to an OD_600_ of 0.5. They were serially diluted and spotted on the plates (one containing synthetic media lacking histidine and uracil and other with the same components but with supplemented 5-FOA). (D) Genomic DNA was isolated from SKCY43 before and after FOA treatment. PCR amplification of *ScTOPOII* resulted in a band of size 4.2 kb. PCR on genomic DNA isolated from SKCY41 strain after 5-FOA treatment confirmed the loss of the *ScTOPOII*-bearing plasmid. However, *Plasmodium* TOPOVIB and *TOPOVIA* could be amplified.

We have generated three *HIS3*-based vectors harboring different versions of PfTopoVI and checked whether they can complement type IIA topoisomerase activity (Fig. 3). The first vector harbors only *PfTOPOVIB*; the second one expresses both *PfTOPOVIB* (as a Myc-tagged protein) and *PfTOPOVIA* (as a Flag-tagged protein) from a bidirectional promoter; and the third one expresses *PfTOPOVIBΔC* and *PfTOPOVIA*. Expression of PfTopoVIB (63 kDa) and PfTopoVIA (35 kDa) was confirmed by Western blot analysis (Fig. 4A).

Our experiment shows that *ΔtopoII* cells transformed with *pScTOPOII* (His^+^) vectors produce 5-FOA-resistant colonies as seen as in Fig. 4B, panel 1. We observed that the *ΔtopoII* cells transformed with *pPfTOPOVIB* vector alone or truncated *PfTOPOVIB* (*PfTOPOVIBΔC*) and *PfTOPOVIA* together failed to grow on the 5-FOA plate, indicating that *PfTOPOVIB* alone cannot complement the function of *ScTOPOII* (Fig. 4B, panels 2 and 4). The results of our yeast two-hybrid experiment showed a strong interaction between *PfTOPOVIBΔC* and *PfTOPOVIA*; however, this interaction was probably not sufficient to rescue the Δ*topoII* lethal phenotype. The *topoII* deletion strain harboring full-length *PfTOPOVIB* and *PfTOPOVIA* together can complement the function of *ScTOPOII* (Fig. 4B, panel 3). In order to understand whether there is any difference between Δ*topoII*/*PfTOPOVIB-VIA* and Δ*topoII*/*ScTOPOII* cells in growth phenotypes, we spotted equal numbers of serially diluted cells before and after the treatment with 5-FOA. Our results show that cells harboring *PfTOPOVIB-VIA* can support the growth of Δ*topoII* cells in similar manners (Fig. 4C). Thus, type IIB topoisomerase (e.g., *PfTOPOVI*) can complement the activity of type IIA topoisomerase (*ScTOPOII*).

To confirm that plasmid harboring *ScTOPOII* (*URA3*) is completely lacking from the Δ*topoII*/*PfTOPOVIB-VIA* strain, we performed PCR analysis of the three samples as shown in Fig. 4D. The figure shows the presence of a 4.2-kb *ScTOPOII* amplified band before the 5-FOA treatment. In the Δ*topoII*/*ScTOPOII* strain, the same band was amplified after the 5-FOA treatment. However, such a band was absent in the 5-FOA-treated Δ*topoII*/*PfTOPOVIB-VIA* strain. Additionally, two bands corresponding to 1.686 kb (*PfTOPOVIB*) and 0.984 kb (*PfTOPOVIA*) were observed. Thus, our experiments demonstrated that *PfTOPOVIB* and *PfTOPOVIA* together were able to functionally complement the activity of *ScTOPOII*.

### PfTopoVIB can decatenate DNA in an ATP- and magnesium-dependent manner.

**FIG 5:**
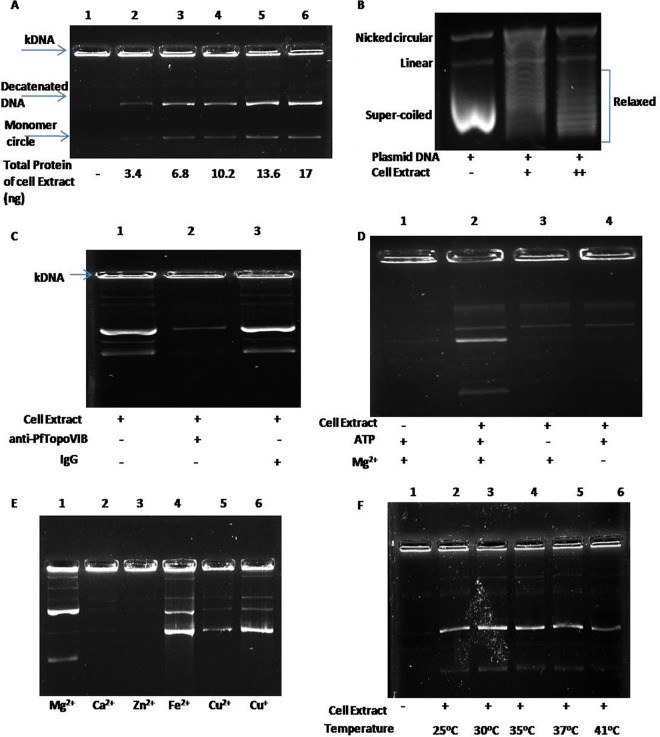
PfTopoVIB can decatenate DNA in an ATP- and magnesium-dependent manner. (A) PfTopoVI-mediated decatenation activity is demonstrated. The catenated kinetoplast DNA (k DNA) remains in the well in the absence of the cell extract which serves as a negative control (lane 1). The decatenated monomer circles are formed by incubating the cell-free extracts harboring PfTopoVIB and PfTopoVIA and can enter the gel (lanes 2 to 6). (B) The cell extract harboring PfTopoVI can relax super-coiled DNA in a dose-dependent manner. (C) Addition of PfTopoVIB-specific antibody for 2 h causes inhibition of PfTopoVI-dependent decatenation activity (lane 2). However, the cell extract-mediated decatenation is not altered in the presence of control IgG (lane 3). (D) The cell extract harboring PfTopoVI is unable to perform decatenation in the absence of either ATP (lane 3) or magnesium (lane 4). (E) The reaction mixtures containing a 10 mM concentration of various divalent and monovalent cations were individually subjected to decatenation reaction. The cell extract containing PfTopoVIB was able to utilize Mg^2+^, Fe^2+^, Cu^2+^, and Cu^+^ as cofactors (lanes 1, 4, 5, and 6). However, it was unable to utilize Zn^2+^ and Ca^2+^ as cofactors (lanes 2 and 3). (F) Temperature-dependent decatenation activity of cell extract harboring PfTopoVI. The levels of dacatenation activity were similar in the temperature range of 25°C to 37°C (lanes 2, 3, 4, and 5); however, the activity was moderately reduced at 41°C (lane 6).

In order to evaluate the activity of PfTopoVI, we prepared the crude cell extract which overexpresses both PfTopoVIB and PfTopoVIA from inducible promoters. As continuous overexpression of topoisomerase II is deleterious to the cell (15), we induced the enzyme for a shorter time period and isolated total protein from the crude extract. Using Bradford reagent, we measured the total protein content and used different concentrations of proteins for performing the decatenation reaction. Our results show that as little as 3.4 ng of the total protein extracted from the induced cell can decatenate DNA (Fig. 5A) and that the amount of decatenated DNA is increased with increasing concentrations of total cellular proteins. We have also tested whether the cell extract harboring PfTopoVI can relax supercoiled DNA. Our study results show that relaxation of supercoiled DNA caused by PfTopoVI occurs in a dose-dependent manner (Fig. 5B).

The yeast topoisomerase I present in the crude cell extract was able to exhibit DNA relaxation activity; however, it does not possess decatenation activity. Thus, any decatenation activities observed in our assays were exclusively due to the presence of PfTopoVI. To confirm whether this activity was specifically due to PfTopoVI, we incubated the cell extract with PfTopoVIB-specific antibody (13) for 2 h and used it for our decatenation assay. We observed that the enzyme activity was significantly reduced after treatment with the antibody (Fig. 5C). However, the activity was not altered by incubating with the cell extract with IgG for the same interval of time. The decatenation activity of PfTopoVI was found to be ATP and magnesium dependent. Our work showed that when ATP or magnesium chloride was removed from the reaction buffer, the cell extract lost the decatenation activity (Fig. 5D, lanes 3 and 4, respectively). In order to understand the cation requirement of PfTopoVI, we have used various divalent as well as monovalent cations that are present in human serum. Our results show that Mg^2+^, Fe^2+^, and Cu^2+^ as well as Cu^+^ can act as a cofactor of PfTopoVI (Fig. 5E). However, the extents of decatenation are different among the various cations. Mg^2+^ can form monomers, an ability absent in the cases of Fe^2+^, Cu^2+^, and Cu^+^. PfTopoVI cannot catalyze decatenation using Zn^2+^ or Ca^2+^ as a divalent cation. The *P. falciparum* parasite life cycle is completed in two different hosts, namely, human and mosquito, where it adjusts to survive at two distinctly different temperatures. While the normal temperature of humans is 37°C, the temperature inside the mosquito midgut is 27°C. Also, during fevers in humans, the temperature is raised to as high as 41°C. We measured the activity of PfTopoVI at various temperatures and observed that PfTopoVI showed similar activities throughout 25°C to 37°C and that the activity was moderately reduced at 41°C (Fig. 5F). The results of our experiment showed that its decatenation activity is completely diminished at 42°C (data not shown).

### Radicicol and etoposide inhibit the decatenation activity of PfTopoVI.

**FIG 6:**
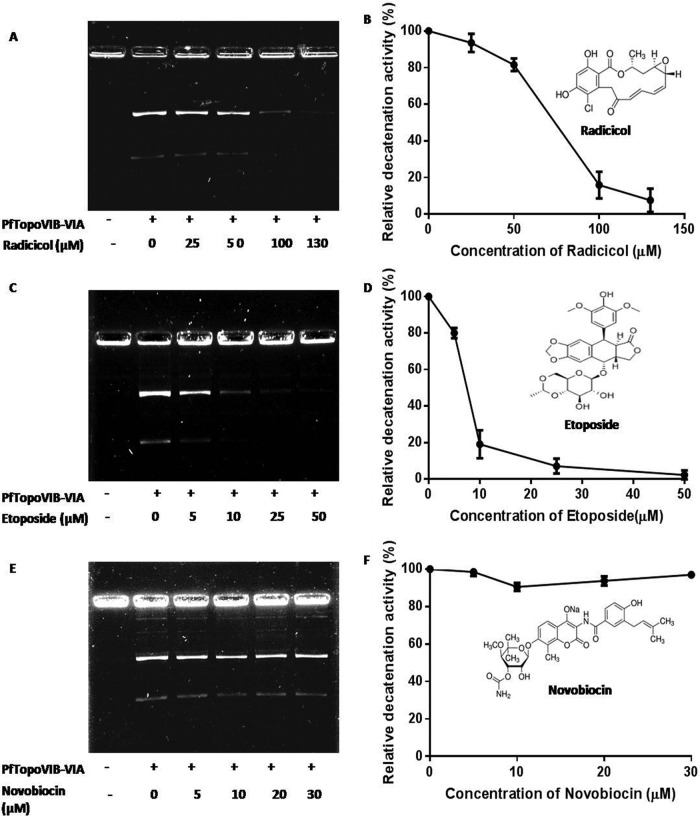
Radicicol and etoposide inhibit the decatenation activity of PfTopoVI. (A, C, and E) Effects of radicicol, etoposide, and novobiocin on PfTopoVI-mediated decatenation activity. (B, D, and F) Graphical representations of the relative amounts of DNA decatenated by PfTopoVI in the presence of the respective inhibitors. The band intensity was measured using ImageJ software. Each point represents the mean value ± SD. The chemical structures of the inhibitors are shown in the inset.

Type II topoisomerases can be inhibited by two mechanisms. One type of inhibitor competes with the ATP binding site of topoisomerase and thereby causes inhibition of its catalytic activity. It has been shown that radicicol binds to the ATP binding pocket (Bergerat fold) of topoisomerase VIB in *S. shibatae* (11). However, members of the coumarin group of compounds such as novobiocin can bind to the GHKL motif that is present in topoisomerase IIA or gyrase but cannot bind to the Bergerat fold. We have taken these two inhibitors and studied their effect on the decatenation activity of the cell extract overexpressing PfTopoVI. We observed that radicicol causes inhibition of PfTopoVI activity in a dose-dependent manner, whereas novobiocin has no effect (Fig. 6A and C). We observed that about 80% of the activity of the enzyme is inhibited at a 100 µM radicicol concentration, whereas the enzyme remains active even in the presence of 30 µM novobiocin. The type II topoisomerase poison etoposide also inhibits the decatenation activity of PfTopoVIB. We observed that 10 µM etoposide can inhibit more than 80% of the decatenation activity of PfTopoVIB.

## DISCUSSION

In recent years, it has been established that topoisomerase VI is involved in endoreduplication, chromatin organization, and transcriptional silencing (16). Our previous study suggested that PfTopoVIB may support schizogony in the erythrocytic stage of the parasite, when endoreduplication occurs (13). Quantitative analysis revealed that *PfTOPOVIB* is expressed at a level 60-fold higher in the schizont stage than in the ring stage (13). This led us to speculate that PfTopoVIB might be important at the specific developmental stage of the parasite when DNA replication takes place.

Although the genome sequence of *P. falciparum* was reported a decade ago, functional characterization of this protein complex was not reported, due to the difficulty in its expression and purification. We tried to express both PfTopoVIB and PfTopoVIA in various bacterial expression vectors but were unsuccessful. However, we could successfully express PfTopoVIB and PfTopoVIA in *S. cerevisiae* using a multicopy yeast expression vector, which allowed its functional analysis for the first time.

Using a genetic approach, we have demonstrated that both subunits of PfTopoVI are necessary for complementation of the yeast *topoII* null mutant. Our complementation assays showed that *PfTOPOVIB* alone is insufficient to complement the function of *ScTOPOII*. Although there is considerable homology between the CAP and TOPRIM domains of *PfTOPOVIA* and *ScSPO11*, our experimental observations suggest that PfTopoVIB probably cannot form a functional complex with ScSpo11 and thus that it is unable to perform complementation. It was reported earlier that TopoVIB of *S. shibatae* interacts with the CAP domain of *S. shibatae* TopoVIA (SsTopoVIA) (9) through its transducer domain. In order to understand whether PfTopoVIB also interacts with PfTopoVIA through its C-terminal domain, we generated several deletion constructs of PfTopoVIB. Our yeast two-hybrid assay results denoted that the C-terminal domain of PfTopoVIB alone cannot interact with PfTopoVIA; however, full-length PfTopoVIB as well as the C-terminal deletion mutant of PfTopoVIB (ΔCPfTopoVIB) strongly interacts with PfTopoVIA. However, results of a genetic complementation study suggest that although the two parts of the truncated protein complex (ΔCPfTopoVIB and PfTopoVIA) showed strong interaction in yeast two-hybrid assays, they are unable to manifest the type II topoisomerase function in yeast. Thus, the C-terminal domain of PfTopoVIB is critical for the functional activity of PftopoVI.

The three-dimensional structure of the C-terminal domain of PfTopoVIB might be different from that of archaeal and plant orthologs. A multiple-sequence-alignment study also revealed that the transducer domain is absent in PfTopoVIB although there exists considerable homology in the core ATPase domain within its orthologs. Thus, future study is needed to understand the three-dimensional structure of PfTopoVI.

Using a yeast cell extract, we have established a biochemical assay system that can be exploited to screen inhibitors of PfTopoVI. In our assay, as little as 3.4 ng of total protein can be used to perform detectable decatenation of DNA. Thus, our assay system is a very simple and economical platform for screening PfTopoVI inhibitors. As proof of concept, we have shown that the topoisomerase poison etoposide as well as radicicol significantly inhibits the decatenation activity of PfTopoVI. While etoposide is a generalized type II topoisomerase inhibitor, radicicol specifically inhibits topoisomerase VIB. Our earlier result showed that radicicol can dock to the Bergerat fold present in the ATPase domain of PfTopoVIB (13). The current study established experimentally that radicicol can inhibit the decatenation activity of PfTopoVI.

We found the 50% inhibitory concentration (IC_50_) of radicicol in cell extract harboring PfTopoVI is around 80 µM. In parasite culture assays, radicicol showed a much lower IC_50_ of around 8 µM (13). The apparent discrepancy in the IC_50_s could be explained by the fact that the gel-based decatenation assay requires a high concentration of kinetoplast DNA (kDNA) to be visualized in an ethidium bromide gel. Thus, the amount of PfTopoVI required in the *in vitro* biochemical assay is also considerably higher than its amount inside the parasite.

In summary, we have demonstrated that PfTopoVI is a type II topoisomerase which decatenates catenated DNA in an ATP- and magnesium-dependent manner and that it can be inhibited specifically by radicicol. Thus, our present work complements our previous study (13) and shows that chemical knockout of *PfTOPOVIB* by radicicol leads to inhibition of mitochondrial replication and schizont arrest which is lethal to the parasite. In this way, the present study has underscored the status of PfTopoVIB as an antimalarial target since this enzyme is absent in human. Additionally, we have established a yeast-based assay system that can be employed for screening various drugs against PfTopoVIB in a faster and more economical manner that can ultimately lead to the discovery of a potent antimalarial compound.

## MATERIALS AND METHODS

### Plasmids.

All PCR primers used in this study are tabulated in Table 3. Using a *P. falciparum* 3D7 cDNA library as a template, we have amplified full-length *PfTOPOVIB* using OMKB51 and OMKB52 as forward and reverse primers, respectively. Also, using the same template, we have amplified full-length *PfTOPOVIA* using OMKB49 and OMKB50 as forward and reverse primers, respectively. The amplified products were cloned individually into *PCR2.1TOPO* (Invitrogen) vector to generate *pcr2.1PfTOPOVIB* and *pcr2.1PfTOPOVIA*. We used *pGBDU-C1* as the bait vector and *pGAD-C1*, which are 2µ plasmids that have uracil and leucine markers, respectively (17), as the prey vector for our assays. *PfTOPOVIB* and *PfTOPOVIA* were subsequently subcloned into *pGBDU-C1* and *pGAD-C1* vectors. For generation of truncated *PfTOPOVIB* that has an ATPase domain, we have amplified the template *pcr2.1PfTOPOVIB* using OSB258 as a forward primer and OSB259 as a reverse primer. Similarly, using the same template, we have amplified *PfTOPOVIBΔC* using OSB33 and OSB239 as forward and reverse primers, respectively. We have also amplified *PfTOPOVIBΔN* using OSB237 and OSB77 as forward and reverse primers, respectively. *PfTOPOVIB* (ATPase), *PfTOPOVIBΔC*, and *PfTOPOVIBΔN* were subsequently cloned into the bait vector with an N-terminal *GAL4* DNA binding domain to generate full-length as well as truncated *PfTOPOVIB*-*BD* fusions. We have also amplified *PfTOPOVIA* with a CAP domain using *pcr2.1PfTOPOVIA* as a template and using OSB257 and OSB256 as forward and reverse primers, respectively. The amplified fragment was subsequently cloned into the prey vector using an N-terminal *GAL4* activation domain to generate a *PfTOPOVIA*(*CAP*)*-AD* fusion. For a yeast complementation assay, we have used two 2µ expression vectors; one has uracil as a marker, and the other has histidine as a marker. The *URA3* marker containing vector *pYES2*/*NTA* (Invitrogen) overexpresses protein as an N-terminal histidine tag, whereas the *HIS* marker containing vector *pESC* (Agilent Technologies) has a bidirectional galactose-induced promoter which overexpresses protein as a C-terminal Myc tag and a C-terminal Flag tag. We have amplified *ScTOPOII* from yeast genomic DNA as a template using OSB265 and OSB266 as forward and reverse primers, respectively. The amplified fragment was cloned into *pYES2*/*NTA*. Similarly, *ScTOPOII* was amplified from yeast genomic DNA using OSB294 and OSB295 as forward and reverse primers, respectively, and cloned into *pESC* (Invitrogen) vector. Full-length *PfTOPOVIB* and *PfTOPOVIA* were amplified using primer pair OSB33 and OSB184 and primer pair OSB129 and OSB185, respectively, with *pcr2.1PfTOPOVIB* and *pcr2.1PfTOPOVIA* as the respective templates. The amplified *PfTOPOVIB* and *PfTOPOVIA* were cloned together in pESC vector, which generated *Myc*-tagged *PfTOPOVIB* and Flag-tagged *PfTOPOVIA*. In a similar fashion, truncated *PfTOPOVIBΔC* was cloned in pESC using OSB33 and OSB239 as a primer pair.

**TABLE 3:** Primers used in this study

Primer	Sequence	Purpose^a^
OMKB51	CACCATGGAAACGTTGAATG	F.P. to amplify *PfTOPOVIB*
OMKB52	CATGATATCATTTATTTC	R.P. to amplify *PfTOPOVIB*
OMKB49	CACCATGCCTCGTCTGGATATC	F.P. to amplify *PfTOPOVIA*
OMKB50	TAAAAGCTCCTTAATGCG	R.P. to amplify *PfTOPOVIA*
OSB258	GACGGATCCACCGGGTTTTATGAAGAAAATGC	F.P. to amplify ATPase domain in pGBDUC1
OSB259	GACGTCGACTTAAGTATGGTTTAAATTTTTATCCATTAC	R.P. to amplify ATPase domain in pGBDUC1
OSB33	GACGGATCCATGGAAACGTTGAATGATAAAAATAAC	F.P. to clone PfTOPOVIB in pESC and pGBDUC1 vector
OSB239	GACGTCGACTTATACACTAGGATTTGTTATAAAATTATTTG	R.P. to amplify *PfTOPOVIB* (*ΔC*) with stop codon cloned in pGBDUC1
OSB237	GACGGATCCATGTTTATAACAAATCCTAGTGTAG	F.P. to amplify ΔN of *PfTOPOVIB* cloned in pGBDUC1 vector
OSB77	GACGTCGACTTACATGATATCATTTATTTCATTTATC	R.P. to amplify ΔN of *PfTOPOVIB* with stop codon cloned in pGBDUC1
OSB257	GACGGATCCATGATATTGTCAAAAGGAAAAATTATAG	F.P. for CAP domain in pGADC1
OSB256	GACGTCGACTTATTCGAAAGCATTCATGCAGTC	R.P. to amplify CAP of *PfTOPVIA* with stop codon cloned in pGADC1
OSB265	GATGGATCCACATGTCAACTGAACCGGTAAGCG	F.P. to amplify *ScTOPOII* cloned in pYES2ANT
OSB266	GACGCGGCCGCTCAATCCTCTTCATTGAACGAAAC	R.P. to amplify *ScTOPOII* cloned in pYES2ANT
OSB294	GATGGATCCATGTCAACTGAACCGGTAAGCG	F.P. to amplify *ScTOPOII* cloned in pESCHIS
OSB295	GACGTCGACATCCTCTTCATTGAACGAAACATC	R.P. to amplify *ScTOPOII* in pESC vector
OSB184	GACGTCGACCATGATATCATTTATTTCATTTATCATTC	R.P. to amplify *PfTOPOVIB* cloned into pESC vector
OSB129	GACGAATTCATGCCTCGTCTGGATATC	F.P. to amplify *PfTOPOVIA* cloned in pESCHIS
OSB185	GACGCGGCCGCTAAAAGCTCCTTAATGCG	R.P. to amplify *PfTOPOVIA* cloned in pESC vector
OSB267	TTTCAGTTAAAGGAGTTTATAACGACCAGCACGGCTAACCCGGATCCCCGGGTTAATTAA	F.P. for *ScTOPOII* knockout
OSB268	ACATATAAAAAGAATGGCGCTTTCTCTGGATAAATATTATGAATTCGAGCTCGTTTAAAC	R.P. for *ScTOPOII* knockout

^a^ F.P., forward primer; R.P., reverse primer.

### Yeast strains.

The strains used in this study are tabulated in Table 4. We used strain PJ69-4A to study yeast two-hybrid interactions (18). As a control, we have cotransformed empty bait vector *pGBDU-C1* and empty prey vector *pGAD-C1* into PJ69-4A to generate SKCY10. The bait-*PfTOPOVIB* (either full-length or truncated) fusion constructs were transformed into PJ69-4A, and the transformants were selected in the medium lacking uracil. Next, we transformed empty prey vector to each one of the strains named above and selected them in the medium lacking uracil and leucine to generate SKCY13, SKCY29, SKCY30, and SKCY37. Also, prey-*PfTOPOVIA* (either full length or truncated with a CAP domain) fusion constructs were individually transformed into the strain either carrying empty *pGBDU-C1* or carrying different bait-*PfTOPOVIB* fusion constructs to generate SKCY15, SKCY36, SKCY17, SKCY24, SKCY25, SKCY32, SKCY33, and SKCY35. To study *PfTOPOVIB* self-interactions, we transformed a prey-*PfTOPOVIB* fusion construct into the cell carrying a bait-*PfTOPOVIB* fusion construct to generate SKCY19. Similarly, to study *PfTOPOVIA* self-interaction, we transformed bait-*PfTOPOVIA* vector into the cell harboring a prey-*PfTOPOVIA* fusion construct to generate SKCY22. *pYES2ANT*/*ScTOPOII* expression vector was first transformed in the W303α strain to generate SKCY38. Using pFA6a-TRP1 plasmid as a template (19), we amplified the *TRP1* cassette with *ScTOPOII* flanking regions using OSB267 and OSB268 as forward and reverse primers, respectively. The product was then integrated into SKCY38 and selected on the medium lacking tryptophan to knock out chromosomal *topoII*. In this way, we generated a SKCY40 strain that harbors *ScTOPOII* from an episomal plasmid. The yeast expression vectors harboring *pESC*/*ScTOPOII*, *pESC*/*PfTOPOVIB*, *pESC*/*PfTOPOVIB-TOPOVIA*, and *pESC*/*PfTOPOVIBΔC-TOPOVIA* were individually transformed to strain SKCY40 to generate strains SKCY43, SKCY45, SKCY41, and SKCY44, respectively.

**TABLE 4:** Yeast strains used in this study

Strain name	Genotype	Source
PJ69-4A	*MATa trpl-901 leu2-3*,*112 ura3-52 his3-200 ga14*Δ *ga180*Δ *LYS2*::*GALl-HIS3 GAL2-ADE2 met2*::*GAL7-lacZ*	17
SKCY10	*MATa trpl-901 leu2-3*,*112 ura3-52 his3-200 ga14*Δ *ga180*Δ *LYS2*::*GALl-HIS3 GAL2-ADE2 met2*::*GAL7-lacZ pGBDUC1 pGADC1*	This study
SKCY13	*MATa trpl-901 leu2-3*,*112 ura3-52 his3-200 ga14*Δ *ga180*Δ *LYS2*::*GALl-HIS3 GAL2-ADE2 met2*::*GAL7-lacZ pGBDUC1*/*PfTOPOVIB pGADC1*	This study
SKCY29	*MATa trpl-901 leu2-3*,*112 ura3-52 his3-200 ga14*Δ *ga180*Δ *LYS2*::*GALl-HIS3 GAL2-ADE2 met2*::*GAL7-lacZ pGBDUC1*/ *PfTOPOVIB*(Δ*C*) *pGADC1*	This study
SKCY30	*MATa trpl-901 leu2-3*,*112 ura3-52 his3-200 ga14*Δ *ga180*Δ *LYS2*::*GALl-HIS3 GAL2-ADE2 met2*::*GAL7-lacZ pGBDUC1*/*PfTOPOVIB*(Δ*N*) *pGADC1*	This study
SKCY37	*MATa trpl-901 leu2-3*,*112 ura3-52 his3-200 ga14*Δ *ga180*Δ *LYS2*::*GALl-HIS3 GAL2-ADE2 met2*::*GAL7-lacZ pGBDUC1*/*PfTOPOVIB* (ATPase) *pGADC1*	This study
SKCY15	*MATa trpl-901 leu2-3*,*112 ura3-52 his3-200 ga14*Δ *ga180*Δ *LYS2*::*GALl-HIS3 GAL2-ADE2 met2*::*GAL7-lacZ pGBDUC1 pGADC1*/*PfTOPOVIA*	This study
SKCY36	*MATa trpl-901 leu2-3*,*112 ura3-52 his3-200 ga14*Δ *ga180*Δ *LYS2*::*GALl-HIS3 GAL2-ADE2 met2*::*GAL7-lacZ pGBDUC1 pGADC1*/ *PfTOPOVIA* (CAP)	This study
SKCY17	*MATa trpl-901 leu2-3*,*112 ura3-52 his3-200 ga14*Δ *ga180*Δ *LYS2*::*GALl-HIS3 GAL2-ADE2 met2*::*GAL7-lacZ pGBDUC1*/*PfTOPOVIB pGADC1*/*PfTOPOVIA*	This study
SKCY24	*MATa trpl-901 leu2-3*,*112 ura3-52 his3-200 ga14*Δ *ga180*Δ *LYS2*::*GALl-HIS3 GAL2-ADE2 met2*::*GAL7-lacZ pGBDUC1*/*PfTOPOVIB* (Δ*C*) *pGADC1*/*PfTOPVIA*	This study
SKCY25	*MATa trpl-901 leu2-3*,*112 ura3-52 his3-200 ga14*Δ *ga180*Δ *LYS2*::*GALl-HIS3 GAL2-ADE2 met2*::*GAL7-lacZ pGBDUC1*/*PfTopoVI*(Δ*N*) *pGADC1*/*pfTOPOVI*	This study
SKCY32	*MATa trpl-901 leu2-3*,*112 ura3-52 his3-200 ga14*Δ *ga180*Δ *LYS2*::*GALl-HIS3 GAL2-ADE2 met2*::*GAL7-lacZ pGBDUC1*/*PfTOPOVIB* (*ATPase*) *pGADC1*/*PfTOPOVIA*	This study
SKCY33	*MATa trpl-901 leu2-3*,*112 ura3-52 his3-200 ga14*Δ *ga180*Δ *LYS2*::*GALl-HIS3 GAL2-ADE2 met2*::*GAL7-lacZ pGBDUC1*/*PfTOPOVIB pGADC1*/*PfTOPOVIA* (CAP)	This study
SKCY35	*MATa trpl-901 leu2-3*,*112 ura3-52 his3-200 ga14*Δ *ga180*Δ *LYS2*::*GALl-HIS3 GAL2-ADE2 met2*::*GAL7-lacZ pGBDUC1*/*PfTOPOVIB* (ATPase) *pGADC1*/*PfTOPOVIA* (CAP)	This study
SKCY19	*MATa trpl-901 leu2-3*,*112 ura3-52 his3-200 ga14*Δ *ga180*Δ *LYS2*::*GALl-HIS3 GAL2-ADE2 met2*::*GAL7-lacZ pGBDUC1*/*PfTOPOVIB pGADC1*/*PfTOPOVIB*	This study
SKCY22	*MATa trpl-901 leu2-3*,*112 ura3-52 his3-200 ga14*Δ *ga180*Δ *LYS2*::*GALl-HIS3 GAL2-ADE2 met2*::*GAL7*-*lacZ pGBDUC1*/*PfTOPOVIA pGADC1*/*PfTOPOVIA*	This study
SKCY38	*MATα leu2-3 112 his3-11 15 ade2-1 trp1 ura3-1 pYES2ANT*/*ScTOPOII*	This study
	*MATα leu2-3 112 his3-11 15 ade2-1 trp1 ura3-1 pYES2ANT*/*ScTOPOII TOPOII*::*TRP*	This study
SKCY43	*MATα leu2-3 112 his3-11 15 ade2-1 trp1 ura3-1 pYES2ANT*/*ScTOPOII TOPOII*::*TRP pESC-HIS*/*ScTOPOII*	This study
SKCY45	*MATα leu2-3 112 his3-11 15 ade2-1 trp1 ura3-1 pYES2ANT*/*ScTOPOII TOPOII*::*TRP pESC-HIS*/*PfTOPOVIB*	This study
SKCY41	*MATα leu2-3 112 his3-11 15 ade2-1 trp1 ura3-1 pYES2ANT*/*ScTOPOII TOPOII*::*TRP pESC − HIS*/*PfTOPOVIA + PfTOPOVIB*	This study
SKCY44	*MATα leu2-3 112 his3-11 15 ade2-1 trp1 ura3-1 pYES2ANT*/*ScTOPOII TOPOII*::*TRP pESC − HIS*/*PfTOPOVIA + PfTOPOVIB*(Δ*C*)	This study

### Yeast two-hybrid analysis.

For yeast two-hybrid analysis, we monitored *ADE2* reporter gene expression as the readout of protein-protein interactions. The cells were grown to an optical density at 600 nm (OD_600_) of 0.5 and then serially diluted as shown in Fig. 2. A 3-µl volume of each diluted cell was spotted simultaneously on two plates; one lacking uracil and leucine and other lacking uracil, leucine, and adenine. Growth on these plates was scored after 5 days of incubation at 30°C. Baits in PJ69-4A were checked for self-activation. The lack of growth ensured that the bait fusions did not lead to self-activation.

### Western blot analysis.

A yeast strain harboring PfTopoVIB-TopoVIA was grown overnight in synthetic complete medium lacking histidine supplemented with 2% galactose. The next day, the cells having total absorbance of 10 (OD_600_) were harvested and proteins were isolated and subjected to Western blot hybridization as previously described (20). The blot was probed with anti-MYC (from Abcam) and anti-Flag (from Sigma) antibodies using 1:5,000 dilutions. Horseradish peroxidase (HRP)-conjugated rabbit IgG (Santa Cruz Biotechnology) and mouse IgG (Promega) were used at a 1:10,000 dilution as secondary antibodies against MYC and Flag, respectively. The blot was developed by using a chemiluminescence detection system (Pierce).

### RNA isolation and RT-PCR.

Total RNA was isolated from SKCY25 and SKCY33 strains using the acid-phenol method (20). cDNA prepared from SKCY25 was amplified using reverse transcriptase along with OSB237 and OSB77 as forward and reverse primers to obtain an 843-bp band corresponding to the C-terminal end of *PftopoVIB*. Similarly, cDNA made from strain SKCY33 was amplified using OSB257 and OSB256 as forward and reverse primers, respectively, to obtain a 300-bp band corresponding to the CAP domain.

### Preparation of cell extract.

Strain SKCY41 carrying *pESC-PfTOPOVIB-PfTOPOVIA* was grown in synthetic complete media lacking histidine and supplemented with 2% (wt/vol) glucose. At the late log phase, the culture was diluted 100-fold in the same medium and continued to grow. When the absorbance of the cells reached 0.75 at 600 nm, 2% galactose (wt/vol) was added to the medium and the cells were grown for 12 h. After that, cells were harvested and resuspended in lysis buffer (50 mM Tris-HCl [pH 7.4], 1 mM EDTA, 1 mM dithiothreitol [DTT], 1 mM phenylmethylsulfonyl fluoride [PMSF], 10% glycerol). Then, glass beads were added to the cells and the reaction mixture was subjected to vortex mixing for 10 15-s bursts, with intermediate cooling for 1 min at 0°C. The mixture was then centrifuged at 12,000 rpm for 15 min to remove the cell debris. The supernatant containing the cell-free extracts was collected and the total protein estimated by Bradford assay, taking bovine serum albumin (BSA) as the standard.

### Decatenation and relaxation assay for DNA topoisomerase.

Analysis of the decatenation activity was carried out by using kinetoplast DNA (Topgen) as a substrate. The reaction was performed with the optimum concentration of cell extract in a reaction buffer containing 125 ng of kinetoplast DNA, 20 mM Tris-HCl (pH 7.5), 1 mM EDTA, 1 mM DTT, 1 mM ATP, 10 mM MgCl_2_, 150 mM KCl, and 0.003% BSA. The reaction mixture was incubated at 30°C for 30 min and was electrophoresed in a 1.0% agarose gel for 2 to 3 h at 5 V/cm. The gel was stained with ethidium bromide for 20 min and then destained with water. Finally, gel photography was performed using a gel documentation system (Protein Simple; Alpha Imager).

The relaxation assay was carried out with 600 ng of supercoiled DNA in a reaction buffer containing 50 mM Tris-HCl (pH 7.4), 1 mM DTT, 0.5 mM ATP, 5 mM MgCl_2_, and 165 mM KCl. The reaction mixture was incubated at 30°C for 30 min. After that, the reaction was terminated by heating at 65°C for 10 min. Samples were subjected to 1% agarose gel electrophoresis at 20 V for 12 h. After electrophoresis, the gel was stained with ethidium bromide and subsequently destained and photographed as mentioned above.

### Drug sensitivity assay.

In a typical drug sensitivity assay, the reaction mixtures containing cell extract and kinetoplast DNA were incubated with a particular concentration of drug in the same manner as described above. Etoposide, radicicol, and novobiocin were individually dissolved in dimethyl sulfoxide (DMSO), ethanol, and water, respectively. Decatenated DNA products were quantified using ImageJ software. The relative band intensities were plotted against various doses of inhibitors. Each experiment was repeated at least three times, and the mean values (± standard deviations [SD]) of the results of three independent experiments were plotted against increasing concentrations of the respective drugs using GraphPad prism 6 software.
